# Case of catheter ablation of atrial fibrillation in a very young patient without structural heart disease

**DOI:** 10.1002/ccr3.5623

**Published:** 2022-03-18

**Authors:** Kohei Sawasaki, Natsuko Hosoya, Masahiro Muto

**Affiliations:** ^1^ 37050 Department of Cardiology Hamamatsu Medical Center Shizuoka Japan

**Keywords:** atrial fibrillation, catheter ablation, pulmonary vein isolation, very young age

## Abstract

The patient was an 18‐year‐old man who suffered frequent supraventricular premature complexes (SVPCs) and atrial fibrillation. Catheter ablation was performed, and the left pulmonary vein had been isolated, although firing from within the left inferior pulmonary vein remained. After that, the patient did not exhibit SVPCs and atrial fibrillation.

## Graphical abstract

Regardless of patient age, the pulmonary vein is highly likely to be the source of arrhythmogenic activity causing atrial fibrillation. Currently, it is possible to radically cure atrial fibrillation with ablation via pulmonary vein isolation.

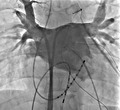

## 1 INTRODUCTION

It is known that as an individual age, atrial fibrillation increases in frequency. Currently, it is possible to radically cure atrial fibrillation with ablation via pulmonary vein isolation. However, the efficacy of ablation of atrial fibrillation in very young patients is not fully understood.

## 2 CASE REPORTS

**FIGURE 1:**
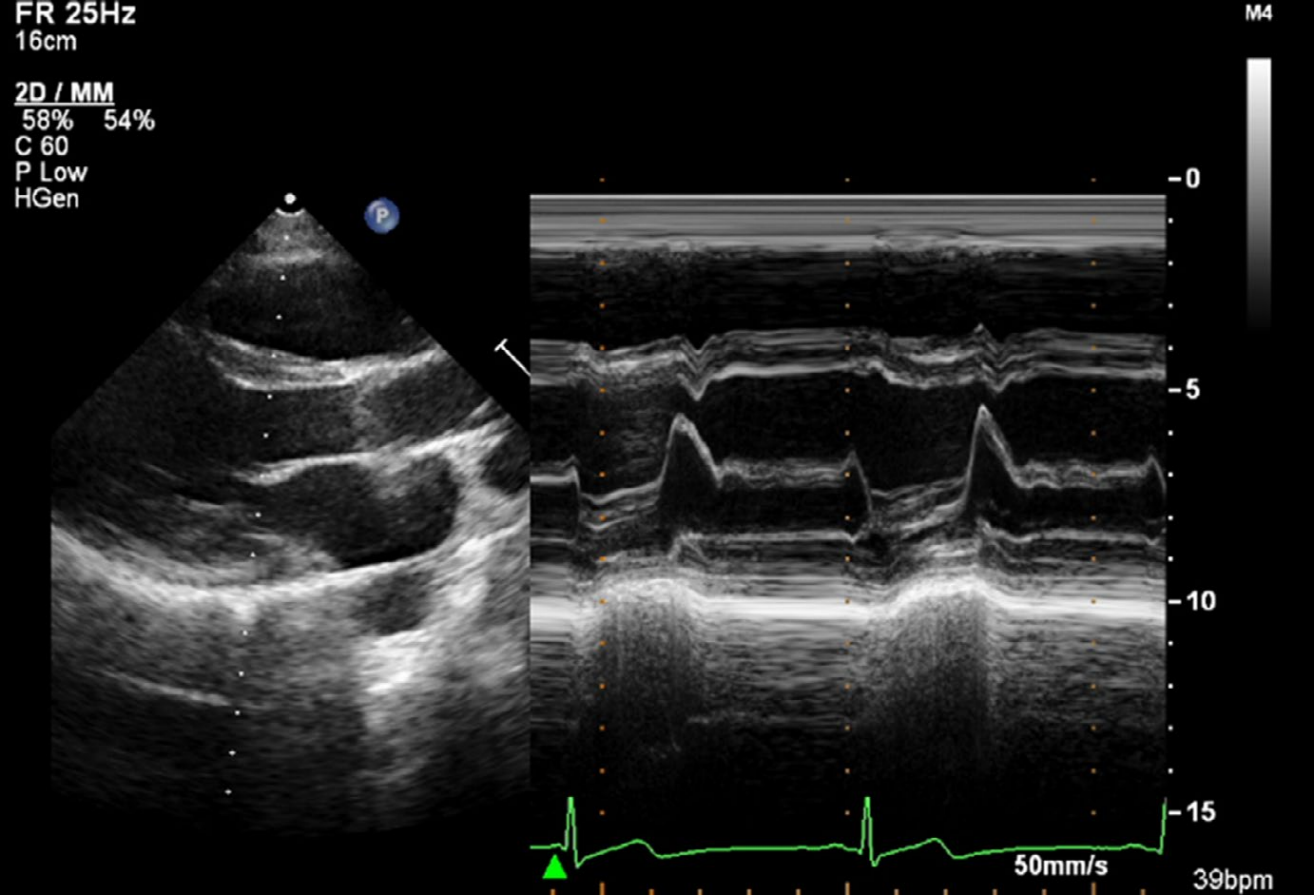
Shows echocardiography. No structural heart disease is observed

**FIGURE 2:**
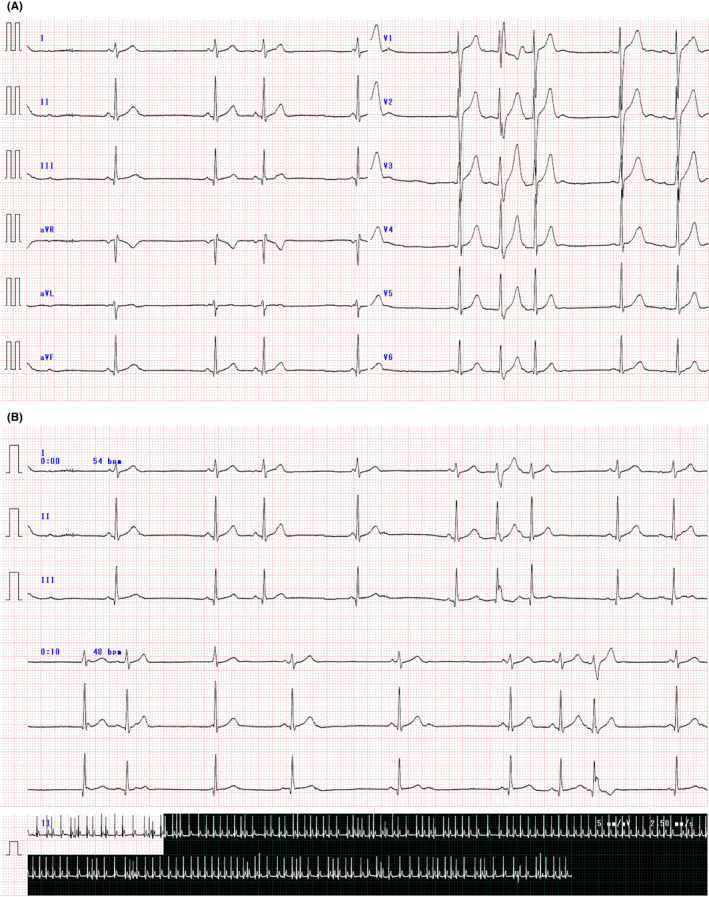
Shows ECG. (A) 12 leads ECG, (B) 3 min ECG. Frequent SPVCs are observed

The patient was an 18‐year‐old man. The patient's medical and family histories were unremarkable. He received no oral medications. A school health check‐up performed when the patient was 15 years old revealed supraventricular premature complexes (SVPCs). However, no symptoms of palpitations were observed. The patient health status was observed by a local doctor. Although echocardiography showed no structural heart disease (Figure 1), Holter ECG revealed frequent SVPCs. Therefore, the patient underwent follow‐ups for 1 year afterward. A Holter ECG performed when the patient was 17 years old revealed approximately 4‐h atrial fibrillation episodes. Moreover, frequent SVPCs were observed at other times (Figure 2). The patient was referred to our hospital for treatment and catheter ablation was performed. As an anticoagulant agent, rivaroxaban (15 mg) was administered 1 month before ablation. No antiarrhythmic drugs were administered.

**FIGURE 3:**
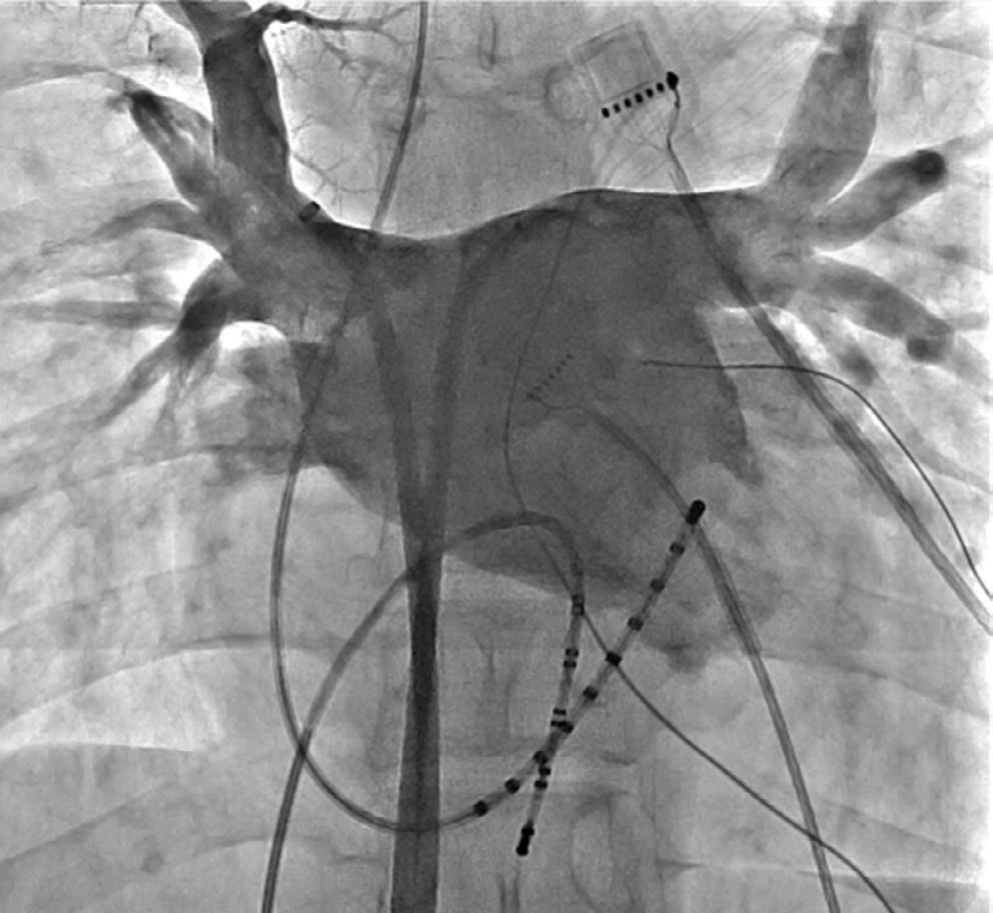
Shows pulmonary venography. No specific finding is observed

**FIGURE 4:**
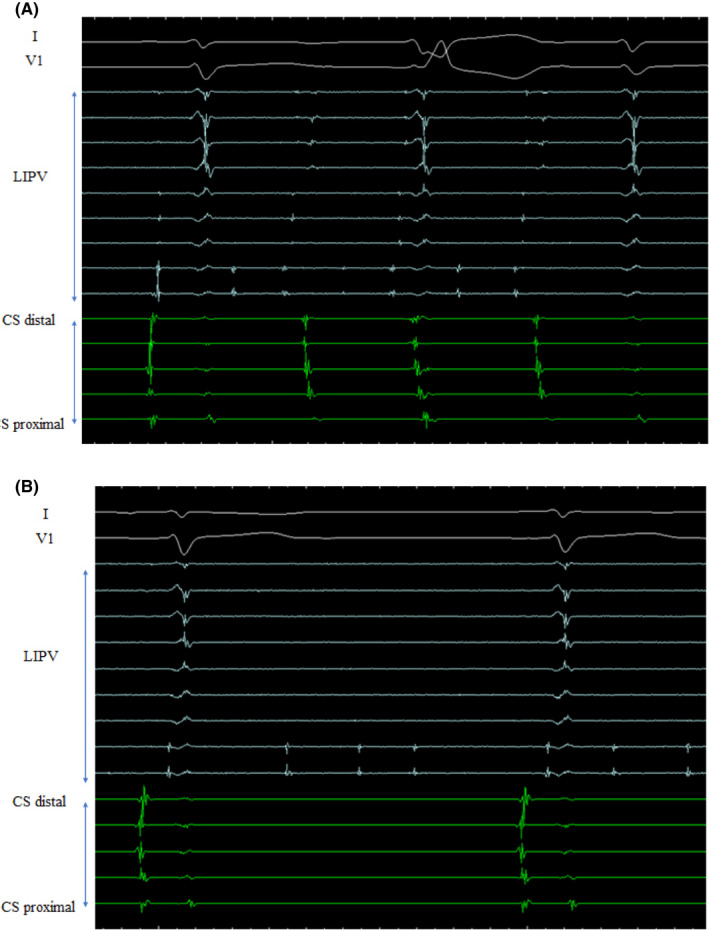
Shows intracardiac electrogram. (A) Before performing extensive encircling pulmonary vein isolation (EEPVI), firing is observed within the left inferior pulmonary vein. (B) While performing extensive encircling pulmonary vein isolation, the patient's SVPC disappears once the left pulmonary vein is isolated. However, firing within the left inferior pulmonary vein remained

At the start of the procedure, a Bee AT catheter (Japan Lifeline Co., Ltd) was advanced into the distal coronary sinus (CS) through the right subclavian vein. The distal and middle eight poles were positioned in the distal CS and right atrial lateral wall, respectively. Surface electrocardiogram (ECG) and bipolar endocardial electrograms were continuously monitored, and the ensuing data were stored on a computer‐based digital amplifier (Labsystem Pro, Bard Electrophysiology). After trans‐septal puncture, 10‐polar circular electrode catheters (Lasso, Biosense Webster), and an irrigation catheter (Thermo‐cool, Biosense Webster) were advanced into the left atrium (LA) through the long SL0 sheaths (St. Jude Medical). Pulmonary venography showed no specific findings (Figure 3). Firing within the left inferior pulmonary vein (LIPV) was revealed by 10‐polar circular electrode catheters (Figure 4). Intraprocedural anticoagulation for catheter ablation was achieved with heparin at doses that maintained activated clotting time at >250–300 s. During performing extensive encircling pulmonary vein isolation (EEPVI), once the left pulmonary vein had been isolated, the patient's SVPCs disappeared. However, firing within the left inferior pulmonary vein remained (Figure 4). No dormant conduction was observed. Bilateral pulmonary vein isolation was confirmed. The absence of any complications, such as pericardial fluid retention, was confirmed, and catheter ablation was performed. Subsequent outpatient follow‐ups were performed, administration of rivaroxaban was continued until 2 months after ablation, and terminated after confirming that sinus rhythm was maintained. Over more than 1 year, the patient's sinus rhythm did not exhibit SVPCs.

## 3 DISCUSSION

Haïssaguerre et al^1^. reported that the pulmonary veins are an important source of ectopic beats, initiating frequent paroxysms of atrial fibrillation. In this case, ectopic beats originating in the left inferior pulmonary vein were observed. After ablation, once the left pulmonary vein had been isolated, the patient's SVPCs disappeared. The patient's sinus rhythm has remained steady since.

Atrial fibrillation has been reported to occur more frequently with age.^2^ The average age of patients with atrial fibrillation has been reported to be 78.9 years. However, the patient, in this case, was very young.

Several reports on atrial fibrillation in young individuals have been published. Gourraud et al.^3^ reported that atrial fibrillation in young patients could reveal genetic pathology or be the initial presentation of cardiomyopathy. In this case, no findings suggestive of genetic pathology or cardiomyopathy were observed. However, we should continue to closely follow up this patient.

In this case, the patient was very young. Therefore, it was considered to limit ablation to the culprit pulmonary vein (LIPV) rather than performing extensive encircling of all PVs. However, since it cannot be denied that firing may appear in other PVs in the future, ablation was performed in all PVs.

Saguner et al.^4^ stated that catheter ablation of atrial fibrillation is effective in very young adults and associated with an acceptable complication rate. In our case, catheter ablation was exceedingly effective, and the patient's prognosis was good. However, the patient, in this case, was even younger than that reported by Saguner et al. No reports of such a young patient have been published before.

## 4 CONCLUSIONS

We report successful catheter ablation of atrial fibrillation in a very young patient without structural heart disease. In this case, the source of the atrial fibrillation was the pulmonary vein, and the usual catheter ablation procedure was effective in radically treating this patient's atrial fibrillation. These results suggest that regardless of patient age, the pulmonary vein is highly likely to be the source of arrhythmogenic activity that could initiate atrial fibrillation. In addition, pulmonary vein isolation is the first‐choice treatment for this condition.

## CONFLICT OF INTEREST

None.

## AUTHOR CONTRIBUTIONS

KS contributed to the conception and design of the study and data analysis and interpretation and manuscript writing. NH and MM contributed to data analysis and interpretation. All authors participated in the collection and/or assembly of data. All authors read, revised, and approved the final manuscript.

## ETHICAL APPROVAL

This study was conducted according to the principles of the Declaration of Helsinki.

## CONSENT

Written informed consent was obtained from the patients.

## Data Availability

Research data are not shared.
